# 

**Published:** 1899-10-07

**Authors:** 


### The Hospital

43

A JOURNAL OF

THE MEDICAL SCIENCES AND HOSPITAL ADMINISTRATION,

INCLUDING

A SPECIAL SECTION FOR NURSES.

EDITED BY

SIR HENRY BURDETT, K.C.B.,

AND

SOLOMON C. SMITH, M.D.

IN TWO VOLUMES ANNUALLY.

LIBRARY

SURGEON GENERAL'S OFFICE

FEB. 11.-1901

168990.

VOLUME XXVII.

October, 1899, to March, 1900.

London:

PUBLISHED BY THE SCIENTIFIC PRESS (LIMITED), AT THE HOSPITAL BUILDING,
28 & 29, SOUTHAMPTON STREET, STRAND, W.C.

MDCCCC.

W1 H069X

### INDEX TO VOL. XXVII., 1899—1900.

*⁎* Articles under the general heading *Hospital Clinics and Medical Progress* are distinguished by the initial (C.) for *Hospital Clinics*, and by (P.) for the rest of the series. (W.) distinguishes the *Institutional Workshop* articles, and (A.) the *Annotations*.

Abdominal Palpitation (P.), 258
Abdominal Surgery (P.), 195
Abortion, Inciting to Procure, 157
Abortion, Treatment of (P.), 227
Abscess, Sub-phrenic (P.), 342
Abscesses (P.), 114
Achondroplasia, Cretinism and Mongolian Imbecility (P.), 416
Addenbrooke's Hospital (W.), 314, 351
Adiposis dolorosa (C.), 192; (P.), 415
Aërated Waters, 103
Agraphia (P.), 113
Albuminuria (P.), 47, 379
Albumosuria (P.), 46
Alcohol, The Prescription of (A.), 253
Ametropia, Correction of, in Treatment of Trachoma (P.), 260
Anæmia Necrosis of the Intestine (P.), 399
Anæsthetics and Insanity (C.), 110
Aneurism of the Aorta (C.), 177; (P.), 259
Angio-Sarcoma (P.), 293
Ankylosis of Both Temporo-maxillary Joints (C.), 7
Anorexia (P.), 79
Anti-Plague Inoculation (C.), 358
Antiseptic Instinct, The, 88
Antiseptics (P.), 343
Antiseptics, Intestinal (C.), 93
Antitoxin Serum (C.), 75
Antivivisection (A.), 427
Aortic Aneurism (C.), 177; (P.), 259
Aphasia and Testamentary Capacity (W.), 348
Appendicitis (P.), 115; (C.), 142, 193
Appendicitis, The Treatment of, 268
Appendix, Surgery of the (P.), 196
Army Medical Department, The, 407
Army Medical Service, Our Undermanned (A.), 237
Arterio-Sclerosis (P.), 258
Arthrectomy of the Knee-Joint (C.), 273
Arthritis (P.), 162
Arthritis, Rheumatoid (P.), 194
Artificial Feeding of Infants, The (C.), 411
Ascites of Cirrhosis, Treatment of (P.), 310
Ascites Cured by Operation (P.), 195
Aseptic Operation, Some Details as to (C.), 359
Asthma (P.), 324
Astringents, Intestinal (P.), 129
Ataxia, Acute (P.), 128
Athletics, Effect of, on the Heart (P.), 242
Atrophy, Yellow (P.), 115
Baarn, Holland, New Deaconesses' Home at (W.), 331, 351
Baby Farming (A.), 355
Bacillus (P.). *See p.* 244
Bacteria in Rheumatic States (P.), 245
Bacterial Treatment of Sewage, The (W.), 51
Bacteriology of the Conjunctiva (P.), 245
Bacteriology, Progress in (P.), 244
Baths for the People (A.), 41
Belfast Royal Victoria Hospital, The (W.), 51
Beri-Beri in Asylums, On (C.), 28
Bethnal Green, The New Infirmary for (W.), 401
Birmingham Consultative Institute, The (A.), 390
Bishop Auckland Cottage Hospital (W.), 280
Bladder, The (P.), 14
Board of Guardians Heavily Fined, A (W.), 118
Book World of Medicine (*Books, Magazines, and Pamphlets Reviewed*)
  Allbutt, A System of Medicine, Vol. VIII., 296
  Bentley, The Maintenance of Health in Egypt, 116
  British Pharmacopœia, 1898, Pocket Notes on the, 262
  British Sanatoria for Open-Air Treatment of Tuberculosis, 262
  Carless. *See Rose*
  Clarke. Orthopædic Surgery, 82
  Copnall. The Law Relating to Infectious Diseases and Hospitals, 132
  Dolbear and Lodge. Matter, Ether, and Motion, 166
  Donald. Municipal Year Book for 1900, 418
  Duchershoff (translated by Leppington). How the English Workman Lives, 16
  Evermann. Report on Investigations by U.S. Fish Commission, 214
  Fielding. Faith Healing and "Christian Science," 98
  Fletcher. Architectural Hygiene, 133
  Fry's Royal Guide to the London Charities, 230
  Gadd. Synopsis of the British Pharmacopœia, 1898, 166
  Gillespie. Manual of Modern Gastric Methods, 278
  Grafstrom, Medical Gymnastics, 50
  Hamon. The Universal Illusion of Free Will and Criminal Responsibility, 82
Book World of Medicine (*continued*).
  Harrison. Stone, Prostrate and Other Urinary Disorders, 98
  Heitzmann. Urinary Analysis and Diagnosis, 132
  Hinshelwood. Letter, Word, and Mind Blindness, 312
  Hoblyn. Dictionary of Medical Terms, 278
  Jardine. Hæmorrhages of Pregnancy Parturition and the Puerperium, 278
  Jenkins. The Radcliffe Infirmary, Oxford, 116
  Jenkins. Weather Chart for 1900, 198
  Jenner Institute of Preventive Medicine, Transactions of the, 166
  Kelynack. Pathologist's Handbook, 214
  Larking. Notes on Folkestone, 116
  Leppington. *See Duchershoff*
  Liebreich. Effects of Borax and Boracid Acid, 364
  Lodge. *See Dolbear*
  London University Guide, 1899-1900, 198
  Luff and Page. Manual of Chemistry, 418
  M'Kendrick. Herrmann Ludwig Ferdinand von Helmholtz, 346
  Malarial Fever, Hints for the Prevention of, 313
  Medical Annual Synoptical Index, 246
  Merck's Manual of the Materia Medica, 364
  Murrell. Aids to Materia Medica, 364
  Neale. Medical Digest, 262
  Nunn. Cancer, 214
  Page. *See Luff*
  Pick. Surgery, 230
  Platt. Fractures of the Upper Extremity, 346
  Richet. Dictionnaire de Physiologie, 262
  Rose and Carless. Manual of Surgery, 198
  Shuttleworth. Mentally Deficient Children, 418
  Snell. On the Prevention of Eye Accidents in Trades, 262
  Spencer. Spencer's Disease, 364
  Stedman. Twentieth Century Practice of Medicine, 148, 328
  Thatcher. Common Sense Health Reform, 166
  Thomson. Diseases of the Nervous System, 246
  Thorne. The Schott Methods of Treatment, 16
  Turner. Lord Lister and Surgery, 198
  Unwin's Chap Book, 1899-1900, 346
  Welles. The Lute and Lays, 364
  Who's Who, 1900, 214
  Wilson. Clinical Studies in Vice and Insanity, 246
Boscombe Hospital (W.), 134
Brain, Innervation of Blood Vessels of the (P.), 344
British Medical Association, General Meetings of the (C.), 60
Bromide of Camphor and of Strontium in Epilepsy (P.), 129
Buda-Pest, Hospital of St. John (W.), 117
Bulbar Paralysis (P.), 113
Bullet with Low Velocity (C.), 162
Busy Physician's Public Work, The (A.), 409
Cadet Corps, A (A.), 319
"Cadge of Norwich" (C.), 209
Caffein (C.), 258
Cage Treatment of Superficial Wounds, The (C.), 110
Calomel in Conjunctivitis (P.), 259
Cancer (P.), 179
Cancer, Colloid (P.), 260
Cancer, Mechanical Hypothesis as to the Origin of (C.), 273
Cancer, Origin of (C.), 377
Cancer, Sir William Banks on the Origin of (C.), 394
Cancer of the Uterus (C.), 126
Cataract Extraction, The Evolution of (C.), 77
Celibacy among Working People (A.), 221
Cerebellum (P.), 96
Cerebro-Spinal Meningitis (C.), 375, 393
Cervical Vertebræ, Injuries of the (P.), 97
Charitable Institutions for Special Treatment (A.), 173
Children, Diseases of (P.), 415, 432
Children and Malaria (C.), 431
Chloroform Administration (A.), 3
Chloroform, The Dosage of (C.), 291
Cholelithiasis (P.), 311
Chorley Cottage Hospital (W.), 232
Christmas Season and the Dying Year, The, 203
Civilisation and Obstetrics (C.), 45
Clinical Teaching, Methods of (A.), 41
Clinker and Sludge, 149
Colitis (P.), 145
Colon, Congenital Idiopathic Dilatation of the (P.), 433
Colour Blindness, "The Sun" on (A.), 25
Competing Universities (A.), 409
Compulsory Schooling or Compulsory Education, 17
Conjunctiva, Diphtheria of the (C.), 341
Conjunctivitis (P.), 259
Consultation Hospital, A, 318
Consultative Institute, A (A.), 89
Consulting Physicians for South Africa, 157
Consumption in Asylums (C.), 76
Consumption in Manchester, Fighting (C.), 306
Contagious Disease Among our Soldiers (A.), 189
Convalescents, Branch Hospitals for, 426
Cornual Cells, Integrity of Anterior (P.), 345
Coryza (P.), 324
Country Homes for Working People (A.), 106
Cremation (A.), 221
Cretinism, Sporadic (P.), 275
Crook, The Case of Mrs., 389
Delirium Tremens (P.), 178
Dentists and the War (C.), 226
"Diabetic" Gangrene (C.), 209
Diarrhœa, Epidemic (P.), 229
Digestive Organs, Diseases of the (P.), 79, 94, 114, 129, 145
Dilatation, Congenital (P.), 146
Diphtheria (C.), 8, 27, 43, 59, 74, 209
Diplegia, Cerebral (P.), 416
Diplegia, Facial (P.), 178
Disjointed Profession, A (A.), 25
Doctor's Bill, The (A.), 373
Dorsal Percussion (P.), 325
Double Consciousness (P.), 414
Drainage and Irrigation in Urethrotomy (C.), 413
Drug Rashes (P.), 164
Drugs, The Adulteration of (C.), 44, 93
Drunkenness, Some Strange Forms of, 263
Duodenal Perforation (P.), 399
Dust in Camps (A.), 337
Dysentery (P.), 129
Dysentery, The Saline Treatment of (C.), 323
Dyspepsia, Septic (P.), 79
Early Rising (A.), 123
Eclampsia (P.), 244
Editor, A Calm, 149
Editor's Letter-Box, 54, 86, 136, 154, 201, 249, 333, 424, 441
Educated Working Woman, The, 99, 133
Education Exhibition, The English, 236
Eel in a Water Pipe, An (A.), 73
Electric Power Distribution (A.), 373
Eltham Cottage Hospital (W.), 264
Endocarditis, Infective (P.), 226
Endowment Funds (A.), 221
Enterocele, Partial (P.), 81
Epilepsy (P.), 128
Epilepsy and Hystero-Epilepsy, Pulse and Temperature in (P.), 128
Epilepsy, Minor, Treatment of (C.), 241
Epileptics, Muscular, Hypotonia in (P.), 128
Epiphora (P.), 33
Epistaxis, Treatment of, by a Sponge Plug (C.), 111
"Erin Go Bragh," 408
Euphtalmine (P.), 48
Fallacy of Statistics, The—The "Impasse" at Brussels, 66
Falmouth Sanitation, 353
Fatal Omen, A (C.), 432
Fatty Heart (P.), 242
Feeble-Minded Children, 371
Feeding of an Army, The, 155
Fever Following Delivery, Treatment of (C.), 76
Fever (P.), 275, 292, 308, 379, 397
Fibroma of the Abdominal Wall (P.), 292
Field Hospital Work (A.), 269
Fistula, Traumatic Jejunal (P.), 398
Food Preservatives, 127, 137, 143, 269, 295
Fractures and Dislocations (of the Spine) (P.), 97
French Factory Legislation, 279
Gall Stones (P.), 114
Gargle, How to (C.), 291
Gasserian Ganglion, Removal of the (P.), 65
Gastric Dilatation, Surgery in (C.), 307
Gastro-Enterostomy (P.), 381
General Medical Council, The, 156
General Surgery (P.), 343
Geneva Convention, The (W.), 68
Genito-Urinary Surgery (P.), 13, 434
Glances at the Hospitals (W.), 103, 153, 233, 283, 299
Goitre, Exophthalmic (P.), 275, 276
Goitre, Operative Treatment of (P.), 276
Gonorrhœal Arthritis (P.), 162
Gonorrhœal Salpingitis, Acute (P.), 147
Gout (P.), 144, 162, 194
Gunshot Wounds of the Abdomen (C.), 61, 195, 225; (P.), 342

Gynæcology, Progress in (P.), 146
Hæmaturia, Intermittent Painless (C.), 307
Hæmopneumothorax (C.), 340
"Hay Fever," Symptoms in So-called (P.), 276
Headache, 92
Headaches (P.), 396
Health and Education, 247
Health and House Warming, 318
Health Resorts, Notes on (W.), 19, 152, 168, 182
Heart and Circulation, Diseases of the (P.), 210, 226, 242, 258
Heart Murmurs, The Causation of Functional (P.), 210
Herbert Hospital, Woolwich. *See p.* 249
Heredity, Laws of (P.), 325
Heredity, Morbid (P.), 211, 325
Hernia (P.), 130
Hernia, Inguinal, Radical Cure of (P.), 80, 130
Hernia, Umbilical, Radical Cure of (P.), 81
Hiccough and its Treatment (C.), 223
High Velocity Bullets (C.), 161
Hip, Congenital Displacement of the (C.), 62
Horse Ambulances for London (A.), 173
Hospital Construction (W.), 18, 67, 100, 117, 134, 150, 232, 264, 280, 400
Hospital Festivals, Meetings, &c. (W.), 21, 84, 135, 185, 200, 218, 298, 315, 331, 349, 366, 383, 402, 421, 437
Hospital, The Flying (W.), 402
Hospital Reform Conference, The (A.), 24; (W.), 34
Hospitals, English and Foreign (W.), 134
Hyperchlorhydria (P.), 94
Hyperpyrexia (P.), 144
Immobilisation of the Flaps after Amputation (C.), 306
Indian Famine, The: Help Required (W.), 350
Infants' Food—Tea, Cheese, and Beer (A.), 3
Influenza and High Death Rates (A.), 303
Influenza, The Mortality from (P.), 308
Influenza and Pneumonia (A.), 252
Insane, The Increase in the Number of the (W.), 330
Insane, The Story of the from Year to Year, 102, 119, 135, 153, 168, 233, 249, 281, 299, 333, 401
Insanitary Pavements, 24
Insanity, The Causes of (C.), 395
Insomnia (P.), 112 (C.) 271
Institutional Questions (W.), 21, 37, 53, 68, 85, 104, 120, 169, 201, 218, 265, 283, 315, 331, 351, 370, 424
Intestinal Obstruction (P.), 115, 129, 417
Intestinal Resection and Anastomosis (P.), 417
Intestines, Small (P.), 129
Intestines, Surgery of the (P.), 196, 398, 417
Intussusception (P.), 399, 433
Iritis (P.), 47
Italian Hospital, Opening of the New (W.), 401
Itching (C.), 110
Kidney, Cystic (P.), 30, 435
Kidney, Diseases of the (P.), 30, 46, 378
Kidney, Exploration of the (P.), 434
Kidney, Movable (P.), 31
Kidney, Sarcoma and Carcinoma of the (P.), 434
Kidney, Tuberculosis of the (P.), 434
Kidneys and Ureters, Surgical Diseases of the (P.), 361
Knee-joint, Excision of the (C.), 126
Knee-joint, Internal Derangement of (C.), 240
Knights Hospitallers, 313
Labour, Operative Treatment of (P.), 227
Laminectomy for Dislocation of the Spine (P.), 97
Landry's Paralysis (P.), 178
Laparotomy for Strangulated Hernia (P.), 131
Laparotomy for Uncontrolled Vomiting (P.), 343
Leaky Water Pipes (A.), 287
Legal Intelligence (W.), 348
Legal Notes (W.), 183, 201
Leicester Guardians, The, 187
Leicester Squabble, The (W.), 281
Lesson of the Winter, A (A.), 391
Liver (P.), 95
Liver, Cirrhosis of the (P.), 114
Liver, Floating (C.), 12
Liver and Gall-Bladder, Surgery of the (P.), 195, 310
Liver, Hydatid of the (P.), 311
Liver, Resection of the (P.), 311
Liver, Syphilis of the (P.), 114
Locomotor Ataxy (P.), 113
London, The Moral Condition of, 302
London, The Reconstituted University of (A.), 352
London Sanitation, 251
London Sewage, 390
London Water (A.), 269
London Water, Impurities in, 204
Look Before You Leap (A.), 373
Lumbar Puncture (P.), 178
Lunacy, Commissioners in, for Scotland; Forty-first Report (W.), 51
Lunatic Asylums of Bengal, The (W.), 119
Lupus (C.), 163
Lynching, The Renewal of, 167
Malaria (C.), 323; (P.), 398
Malaria, Koch on (C.), 46
Malaria, The Prevention of (C.), 412
Mammary Carcinoma, Recurrence of (P.), 180
Manchester St. Mary's Hospital (W.), 150
"Map of Life," The, 56
Massage in the Treatment of Fractures (C.), 305
Mastoid Operation, The Complete (C.), 274
Mauser Bullet Wounds (C.), 322
Meat and Cancer (A.), 57
Meat Inspection (P.), 228
Mediastinitis, Chronic (P.), 243
Medical Acts, Reform of the (C.), 376
Medical Addresses, The, 1
Medical Attendance on the Families of Soldiers and Sailors (A.), 139
Medical Education in London. 205
Medical Organisation (A.), 237
Medical Practice by Commercial Companies (C.), 432
Medical Practice, Sale and Purchase of a (W.), 83, 101
Medical Society, The (C.), 30, 62
Medicine, Pharmacy and Dispensing, 2
Men in Armour (A.), 287
Meningitis (P.), 397
Meningitis, The Diagnosis of (C.), 160
Menopause, The (P.), 146
Menopause, Artificial (C.), 145
Mental Dissolution (C.), 321
Mesentery, Tumours of the (P.), 399
Metropolitan Extension, 39
Micro-Organisms as a Cause of Chronic Disease (C.), 289 *and see p.* 333
Midwives Bill, The, 285 (A.), 391, 425
Midwives, Legislation for (W.), 419, 436
Midwives, The Proper Scope of Legislation for, 336
Midwives, The Question of, Once Again (A.), 139
Military Service and National Health, 335
Milk Inspection in New York (C.), 430
Modern Sociology, 17, 66, 99, 133, 149, 167, 199, 231, 247, 263, 297, 313, 329, 347, 365
Motor Nerves, Relation of, to Tissues of Body (P.), 344
Movable Kidney: A Cause of Hepatic Colic (C.), 241
Municipal Hospitals (A.), 287
Myasthenia (C.), 376
Myopia, The Treatment of High Degrees of (C.), 144
Myxœdema (P.), 275
Necrosis, Acute Fat (P.), 342
Nephritis, Causes of (P.), 31
Nerves, Surgery of the (P.), 64
Nervous Decay, Premature, Sir William Gowers on (C.), 176
Nervous System, Diseases of the (P.), 344, 360, 396, 414
Neuritis, Peripheral Gouty (P.), 179
Neuritis, Peripheral, Treated with Arsenic (P.), 416
Neurology (P.), 95, 112, 129, 178
New Appliances and Things Medical, 14, 33, 49, 65, 81, 115, 132, 147, 165, 196, 213, 229, 245, 277, 345, 363, 381, 399, 435
Night Blindness (C.), 125
1900, 219
Nitro-Glycerine (P.), 258
Nodules (P.), 144
Nomenclature, Fashion in (A.), 303
North London Consumption Hospital (W.), 19
Notes and News, 4, 26, 42, 58, 74, 90, 108, 124, 140, 158, 174, 190, 207, 222, 239, 254, 270, 288, 304, 320, 338, 356, 374, 392, 410, 428, 428
Nottingham General Hospital (W.), 67
Nursing of Our Soldiers: A Serious Difficulty (W.), 248
Obesity, Thyroid Treatment of (P.), 276
Obstetrics, Progress in (P.), 227, 243
Occipito-Posterior Positions (P.), 228
Œdema, Angioneurotic (P.), 163
Œsophagus (P.), 79
Old Wives' Physic (C.), 429
One-Portal System, The (A.), 173
Open-Air Treatment and Its Lessons (A.), 409
"Open-Air" and Tuberculosis, 302
Ophthalmia, Gonorrhœal (C.), 239
Ophthalmia, Gonorrhœal, Abortive Treatment of (P.), 32
Ophthalmia, Sympathetic (C.), 192
Ophthalmology, Progress in (P.), 32, 47, 259, 276
Ophthalmoscope, The, in General Practice (C.), 306
Optic Nerve, Atrophy of the (P.), 277
Overcrowding on Foreign Vessels (A.), 123
Ownership of Sanatoria, The, 172
Paget, The Late Sir James, 220
Pancreatic Cysts (P.), 196
Paralysis Following General Anæsthesia (P.), 415
Paralysis, Juvenile General (P.), 415
Parathyroid Glands (P.), 276
Paris as a School of Manners (A.)., 427
Parotid Gland, Carcinoma of the (P.), 261
Pathological Society, The (C.), 94
Patella, Fractured, Treatment of (C.), 306
Pauperism. Is Pauperism Increasing? 347
Peat-Bog, The Future of the, 365
Pelvic Glands, Deep, Radical Removal of (P.), 342
Pemphigus (P.), 165
Penis, Cancer of the (C.), 256
Pericardial Corns (C.), 290
Pericarditis with Effusion, Treatment of (C.), 192
Pericardium (P.), 242
Peripheral Nerves, Injuries to (P.), 64
Periscope of Dermatology (C.), 163
Peritoneum, Surgery of the (P.), 342
Peritonitis (P.), 146, 342
Peritonitis, Tuberculous (C.), 208
Peroneal Nerve, Subcutaneous Injury to the (P.), 65
Personal Infection in Typhoid Fever (A.), 237
Phlebitis (P.), 144
"Phossy Jaw," 199
Phototherapy (C.), 11
Phthisis (C.), 62, 78
Phthisis and Pneumonia (C.), 339
Plague (A.), 89
Plague in Honolulu (C.), 377
Plague in India (C.), 396
Plague, The Spread of (C.), 359
Plantar Reflex (P.), 96
Plaster of Paris Splints, Some (C.), 91, 109
Pleural Effusions (P.), 309
Pneumonectomy (P.), 309
Pneumonia (P.), 309, 324, (C.) 339
Pneumonia, Case Mortality in (C.), 341
Polyadenomata of the Large Intestine (P.), 261
Post-Mortem Appearances and Pathological Processes, Dr. Savage on the Relation between (C.), 176
Prince of Wales's Fund, The, and St. Mark's Hospital (W.), 350
Prince of Wales's Hospital Fund for London, 86, (A) 204, (W.) 216, 332
"Princess Christian" Hospital, The (W.), 421
Psychiatry, Progress in (P.), 48, 211, 325
Public Mortuaries (A.), 57
Puerperal Fever, Treatment of (C.), 207
Puerperal Infection, Local Treatment of (P.), 213
Puerperal Insanity (C.), 175, 191, 207
Pulsus Paradoxus (P.), 258
Purpura (P.), 162
Pyloroplasty (P.), 381
Pylorus, Congenital Hypertrophy of the (P.), 433
Quinine Amaurosis (P.), 48
R.A.M.C. South Africa Fund, The (A.), 319
Racial Stamina, 24
Radcliffe Infirmary and its Chaplain, The (W.), 366
Radiography, Recent Progress in (C.), 5
Rashes after Enemata (P.), 130
Red Noses (C.), 139
Relief Funds for the War, The, 172
Renal Calculi (P.), 13
Renal Calculus (P.), 361
Renal Tuberculosis (P.), 13
Resection of the Intestine, Results of (C.), 290
Respiratory System, Diseases of the (P.), 309, 324
Respiratory Tract, Diseases of the (C.), 62, 78
Restoration of Function after Section of Nerve Trunks (C.), 340
Restlessness of Old Age (P.), 258
Rheumatism in Acute and Valvular Disease (C.), 414
Rheumatism and Gout (P.), 144, 162, 194
Rickets, Clinical Notes on (C.), 357
Rigors (C.), 160
Royal College of Surgeons of England, The, 2[illegible]7
Royal Medical and Chirurgical Society (C.), 430
St. George's Hospital (W.), 249
St. Mark's Hospital and the Prince of Wales's Fund (W.), 382
Sale of Food and Drugs Act, 1899 (W.), 265
Sale of Poisons (C.), 193
Salvation Army Shelters (A.), 205
Sanatorium Treatment Gone Mad (A.), 355
Sandgate Convalescent Homes, Florence Warden on (W.), 382
Sanitary Authorities, Supine (A.), 337
Scarlatina (P.), 397
Schools and Epidemics (A.), 319
Schools and Swimming Baths, 297
Sclerosis, Insular (P.), 128
Scurvy, Rickets, and Civilisation (C.), 225
Searching out the Secrets of Nature, 55, 86
Seborrhœa of the Scalp, Treatment of (C.), 93
Secrecy of Proceedings at Committees (W.), 348
Selfish Parents—Starving Children, 231
Sensory Tracts of the Central Nervous System (P.), 344
Serum Therapy in Puerperal Fever (C.), 76
Serum Treatment (in Diphtheria) (C.), 75
Shingles (P.), 327
Shock (C.), 143
Sick and Wounded, The (A), 189
Sinusitis, Chronic Frontal, A Note on (C.), 77
Skin Affections met with in Bright's Disease (C.), 413
Skin-Grafting (C.), 144
Sociology. *See Modern Sociology.*
Soldiers in Civilian Hospitals (A.), 253
South Africa, 286
South London, The Rulers of (C.), 127
Spartein (P.), 258
Spas, English and Foreign (C.), 111
Specialist and his Telegrams, The (A.), 123
Spinal Cord, Transverse Section of the (P.), 95
Spine, Chronic Rigidity of the (C.), 208
Spine, Injuries of the (P.), 96
Spine, Surgery of the (P.), 163
Spittoon, The Fashionable (A.), 253
State Medicine, Progress in (P.), 228, 294
Stereognostic Sense, Loss of the (P.), 414
Stomach, Injection of Fluids into the (P.), 381

Stomach, Surgery of the (P.), 196, 380
Stomach Tube in Practical Medicine, The (C.), 29
Street Noises to Church Bells, From (A.), 3
Student of Medicine, The, 22, 54, 70, 86, 104, 120, 136, 154, 170, 202, 218, 234, 250, 266, 284, 300, 316, 352, 370, 388, 442
Subsidised Hospitals (A.), 89
Sulphonal, An Excessive Dose of (C.) 273
Summer Diarrhœa in Children (P.), 432
Sunday Entertainments, 105
Surgery, Abdominal (P.), 195
Surgery of the Appendix (P.), 196
Surgery, General (P.), 343
Surgery, Genito-Urinary (P.), 13, 434
Surgery of the Intestines (P.), 196, 398, 417
Surgery of the Liver and Gall Bladder (P.), 195, 310
Surgery of the Nerves (P.), 64
Surgery of the Peritoneum (P.), 342
Surgery of the Spine (P.), 163
Surgery of the Stomach (P.), 196, 380
Surgery Outside the Hospitals, 354
Surgical Dressings, Tinned (A.), 107
Syphilis, The Aix-la-Chapelle Treatment of (C.), 359
Syphilis Vertebral, and Potts' Disease (P.), 163
Syphilitic Taint in the Ova, Prolonged (C.), 431
Tabes, Epigastric Analgesia in (P.), 128
Tabes, Pathology of (P.), 360
Tabes Dorsalis, The Treatment of (C.), 177
Teetotalism and Philanthropy (C.), 10, and *see p.* 54
Tenements of New York, The (C.), 377
Testicle, Chondro-carcinoma of the (P.), 261
Tetanus (P.), 309
Therapeutical Notes, 12, 181, 196, 213, 327
Therapeutics (Dermatology) (P.), 164
Third Pair of Nerves, Sudden Paralysis of the (P.), 277
Thorne Thorne, The late Sir Richard, K.C.B. (A.), 189
Thoroughness in Surgery, 188
Thyroid, Diseases of the (P.), 275
Tinea Capitis, Treatment of (P.), 162
Tobacco-Amblyopia (C.), 141
Tongue, Geographical (P.), 433
Tonsil, The Pharyngeal (P.), 433
Trachoma, Treatment of (P.), 32
Travel in the Treatment of Diseases of the Nervous System (C.), 224
Truck Acts at Home and Abroad, 329
Tuberculin Test, The (C.), 323
Tuberculosis in Cows (C.), 414
Tuberculosis, The Decline of (C.), 11
Tuberculosis in Mammals and Birds (C.), 109
Tuberculosis, The Open-Air Treatment of, 106
Tuberculosis, Renal (P.), 378
Tuberculosis, The Sanatorium Treatment of (W.), 51
Tuberculosis and Slight Ailments, 71
Tuberculous Convalescent, The (C.), 256
Tumours (P.), 164, 179, 260, 292
Tumours of the Corpus Callosum (P.), 414
Tumours, Inoperable, The Treatment of (P.), 293
Typhoid (P.), 274, 379
Typhoid in India (P.), 309
Typhoid Fever in America (C.), 396
Typhoid Fever, Diet in (C.), 255
Typhoid Fever, Inoculation for (C.), 272
Typhoid Fever, Perforation in (C.), 77
Typhoid Fever, Surgical Treatment of (P.), 417
Typhoid Fever, Treatment of (C.), 142
Typhoid Fever Without Abdominal Symptoms (C.), 143
Ulcer of the Stomach (P.), 80, 94
Ulcers, Perforated Gastric (P.), 380
Underfed Children, 87
University, Functions of a (C.), 12
Unqualified Dispensers, 121
Ureter, The (P.), 13
Uric Acid, 235
Urology (P.), 46
Uterine Fibroids, The Treatment of (C.), 291
Uterus, Cancer of the (C.), 126
Uterus, Fibromyomata of the (P.), 147
Uterus, Malignant Disease of the (P.), 146
Uterus, Rupture of the (C.), 257
Vaccination, A New Method of (C.), 341
Vaccination Officers and Their Duties (W.), 118
Vaginal Cœliotomy (C.), 61
Ventilation, 372
Ventral Hernia, Post-Operative (P.), 131
Vermiform Appendix, Hernia of the (P.), 130
Vesicula Seminalis, Tuberculous, Excision of a (C.), 257
Vienna, Hospitals in (A.), 41
Vital Statistics (P.), 294
Volvulus (P.), 417
Wages of Prosperity, The (C.), 395
Wandsworth and Clapham Union Infirmary Nurses' Home (W.), 18
War, The, 72
War and Fever, 138
War Fund, The, 154
Widal's Reaction, On the Value of (C.), 159
Wild Oats, 122
Winter Clothing (A.), 73
Woes of the Profession, The (A.), 427
Women on Hospital Boards (W.), 215
Working-class Members of Hospital Committees (W.), 420
Working Classes, The Housing of the (A.), 303

### THE SPECIAL SECTION FOR NURSES.

Aberdeen Royal Infirmary, 95
Absent-Minded Giver, The, 212
Across the Seas, 7, 26, 39, 51, 78
Age Limit, The, and the Commercial Element in Nursing, 248
American Hospital Ship, The, 66, 117, 155
An Exodus of Nurses to South Africa, 339
Antiseptics for Nurses, 55, 275
Arrival of the "Princess of Wales" Hospital Ship, 285
Baby's Cord, The Treatment of, 81
Bed Sores, Prevention of, Treatment of, 200, 251
Book and its Story, A, 28, 54, 96, 136, 203, 225, 265, 346
Bristol General Hospital, 95
Bristol Jubilee Convalescent Home, The Opening of the, 107
British Home for Incurables, 133
Burns and Scalds (Dangers of), 302
Chorea Cases, The Nursing of, 118
Christmas Decorations, 120
Christmas Entertainments, 184
Christmas Eve at an Ophthalmic Hospital, 169
Christmas in a Fever Hospital, 157
Christmas in the Hospitals, 171
Christmas Library, In a, 145, 158
Christmas Number, Our, 118, 133
Christmas Shopping, 148
Clinical Charts and How to Keep Them, 273
Colonial Nursing, 132
Colonial Nursing Association, The, 65, 77, 89, 103
Commercial Element in Nursing, The, 234, 248
Commissariat Department, The, 317, 343
Concert by Incurable Children, A, 70
Conversazione at the London Hospital, The, 292
Daddy Nine, 17
Day in My District, A, 290
Death in Our Ranks, 37, 52, 66, 79, 235, 331
Dublin Nurses *en route* to South Africa, The, 289
Echoes from the Outside World, 10, 30, 41, 53, 69, 80, 94, 108, 123, 135, 147, 159, 172, 187, 199, 213, 224, 236, 252, 264, 276, 291, 304, 318, 332, 345
Economics of Good Nursing, The, 286
Essentials for a Sick-Room in Protracted Illness, The, 119
Ethics of Nursing, The, 17
Everybody's Opinion, 15, 31, 43, 57, 71, 82, 97, 109, 124, 137, 149, 161, 177, 201, 214, 226, 240, 255, 267, 278, 292, 306, 319, 333, 347
Eviction of British Nurses from Johannesburg Hospital, The, 104
Examination Questions, 18, 68, 319
Experience in the Sierra Nevada Mountains, An, 250
Fevers of the South-West Coast of Africa, The, 67
Heaven-born Doctor, The, 11
Help the Nurses to Help the Sick, 144
Hospital Reform, 79
Hospital Sketches, 42, 286
How Nurse Lilian Spent Her Holiday, 29
Illinois Training School for Nurses, Chicago, 261
Improvements at the Royal Orthopædic Hospital, The, 91
In the City, March 1st, 1900, 300
Italy, Pictures from, 9
Invalid Children's Aid Association, 330
Jottings on Hospital Work in the United States, 344
Lectures on Medicine to Nurses, 260, 284, 299, 342
Lectures to Nurses, 168, 182, 194, 208, 220, 232, 246
Lectures on Nursing for Probationers, 312, 338
Lectures to Surgical Nurses, 24, 36, 50, 64, 76, 88, 103, 116, 130, 142, 154
Louise Darche: A Modern Nurse and Her Work, 105
Maritzburg, Notes from the Camp at, 143
Matron's Corner, The, 305
Mismanagement at a London Poor Law Infirmary, 183
Miss Dashwood's "At Home," 305
Model Lodging-house for Nurses, A, 12
Nasal Feeding, 237
New Year's Eve at Arezzo, 222
Notes on News from the Nursing World, 1, 21, 33, 47, 61, 73, 85, 99, 113, 127, 139, 151, 165, 179, 191, 205, 217, 219, 243, 258, 269, 281, 295, 309, 321, 336
Notes and Queries, 19, 32, 45, 59, 72, 83, 98, 112, 125, 138, 150, 163, 178, 190, 204, 215, 228, 241, 256, 268, 280, 293, 307, 320, 334, 348
Novelties for Nurses, 13, 31, 38, 79, 133, 170, 266, 286, 300, 316, 326, 342
Nurses' Bookshelf, The, 56, 162, 174, 239, 274
Nurse's First Christmas in Hospital, A, 173
Nurses of Guy's Hospital, The, 6
Nurses of the Imperial Yeomanry Hospital, 233, 253, 263, 277
Nurses of the Royal Free Hospital, 210
Nurses of St. George's Hospital, The, 90
Nurses of To-day, 27
Nursing American Soldiers, 131
Nursing on the "Avoca," 316
Nursing at the Chelsea Infirmary, 314
Nursing in a Durham Colliery Village, 235
Nursing in the Fever Hospitals, 52, 67
Nursing in a Frontier Hospital at Bulawayo, 342
Nursing at the Greek Hospital, Alexandria, 40
Nursing on the Hospital ss. "Spartan," 144
Nursing in India, Private, 315
Nursing Kaffirs in Cape Colony, 51
Nursing at Maritzburg, 313, 340
Nursing at the Military Hospital, Malta, 221
Nursing in Military Hospitals, 238
Nursing in Naval Hospitals, 254
Nursing at Netley, 299
Nursing in the Orkneys, 273
Nursing in Roumania, Private, 134
Nursing in Syria, Private, 237
Nursing in West Africa, 198
Nursing the Wounded in the American Civil War, 197
Nursing the Wounded at Naauwport, 331, 341
Nursing the Wounded at Woolwich, 209
Nursing at Wynberg, 303
Papers for Probationers, 25, 93, 223
Placenta Prævia, Clinical Lecture on Two Cases of, 4
Presentations, 7, 31, 40, 66, 83, 95, 110, 133, 171, 202, 211, 226, 238, 279, 288, 305, 333, 347
Princess Christian Hospital Train, The, 157
Princess of Wales's Nurses for the War, 167
Princess of Wales's Nurses on their Way to South Africa, 301
Princess of Wales's War Fund, The, 160, 175, 188, 196, 212, 237
Proposed Cottage Hospital at Moose Fort, 5
Queen, The, at the Royal Infirmary, Ryde, 277
Queen's Nurses, The, Appointments for 1900, 189
Queen's Visit to Italy, The, 247
Question of Immediate Importance to all Trained Nurses, A, 195
Reading to the Sick, For, 14, 32, 45, 59, 70, 98, 110, 125, 138, 163, 178, 190, 204, 215, 228, 241, 256, 268, 280, 293, 307, 320, 334, 348
Royal British Nurses' Association, 133
Royal National Pension Fund for Nurses, 327
St. George's Infirmary, Fulham Road, 233, 266
Sick and Wounded in the "Winifredian," The, 325
Sidelights on Asylum Nursing, 9
"Sister," The Title of, from a Nurse's Point of View, 326
South Africa as a Field for English Nurses, 37
Steam Disinfection Apparatus, 55
Teeth, Concerning, 57
Temperance, A Youthful Advocate of, 12
To a Nurse, 37
To Nurses, 121
To the Nurses of the Imperial Yeomanry Hospital, 277
Transvaal, The Crisis in the, 11
Travel Notes, 20, 46, 60, 84, 126, 164, 216, 242, 294, 308
"Typhoid" Epidemic, A, and How We Nursed It, 122
Typhoid, A Few Hints for the Private Nursing of, 134
Typhoid Fever, The Dangers of Nursing, 91, 107
Typical Patients, 18
Wardmaids I Have Known, Some, 92
Wedding, 226
Winter Sale of Ladies' Work, 137
Woolwich, New Nurses' Home at, 38
Zenana Bible and Medical Mission, 57

Printed by W. H. and L. Collingridge, City Press, 148 and 149, Aldersgate Street, London.

### NOTICE TO CORRESPONDENTS.

All MS., Letters, Books for Review, and other matters intended for the Editor should be addressed The Editor, "The Hospital," The Hospital Building, 28 & 29, Southampton Street, Strand, W.C.

The Editor cannot undertake to return rejected MS., even when accompanied by stamped directed envelope.

**Notice to Advertisers and Subscribers.**

All Advertisements, Orders for Copies of The Hospital, and Business Communications should be addressed to **THE MANAGER (Not to the Editor),** The Hospital, The Hospital Building, 28 & 29, Southampton Street, Strand, London, W.C.

The Cost of Subscription, post free, is as follows:—Per week, 2½d.; per quarter, 2s. 8d.; per half-year, 5s. 3d.; per annum, 10s. 6d. Foreign:—Per annum, 15s. 2d., payable, in all cases, in advance.

**NOTICE.—Vol. XXVI. of "The Hospital" (April, 1899, to September, 1899), in handsome cloth binding, will shortly be on sale at the Office of this Journal, price 7s. 6d. Cases for binding the Numbers contained in this Volume 1s. 6d. Index to Vol. XXVI., 2½d., post free.**

**The Monthly Part for October is now ready. Price 6d., by post 8d.**

### Notes and News.

### BOOKS RECEIVED.

The Scientific Press.

"From a Nurse's Note-book." By Honnor Morten.

Hadden, Best, and Co.

"The Law Relating to Infectious Diseases and Hospitals." By H. H. Copnall.

Robert Boyle and Son.

"Natural and Artificial Methods of Ventilation."

Skeffington and Son.

"Only Joe; or, Short Tales of Homely Hearths." By J. E. Cutcliffe.

University Press.

"Darwin on Trial." By Democritus.

"The Universal Delusion of Free Will and Criminal Responsibility." By A. Hamon.

"The Pathology of the Emotions." By Ch. Féré. Rendered into English by Robert Pash, M.D.

### INSTITUTIONAL QUESTIONS.

### SCRAPS AND GLEANINGS.

At the present time there is a deficit on the general account of the Hartlepool Hospital of about £600, owing to the regrettable fact that for the past few years the income has remained stationary despite the circumstance that the number of patients has increased, thus swelling the expenditure.

There was an advance this year of about 20 per cent. in the collections at places of worship in Bristol on behalf of the Bristol General Hospital. The legacies during the past year show a decrease of £4,000, but the donations increased by £2,041, which sum, however, includes two donations of £1,000 each.

It is estimated that £20,000 would be required to erect a new hospital for infective diseases for Dublin to accommodate 80 patients. The Lord Mayor of Dublin at a recent meeting insisted upon the necessity for such a hospital, and one or two convalescent homes in connection therewith. It was proposed that the sanitary authorities concerned should "appoint a sub-committee representative of each board or council for the purpose of preparing a scheme and selecting a site or sites; and that a resolution agreeing to bear the expense proportionately of building and maintaining the hospitals should be adopted by each of the bodies concerned." It is expected that the Councils addressed shall send a reply by October 3rd.

During 1898 17,287 patients were treated at the Wolverhampton General Infirmary.

The Health Committee of the Corporation of Blackburn have decided that they cannot see their way to enter into any arrangement for taking into their Bull Hill Hospital any infectious diseases from the Turton district other than small-pox.

At a recent meeting of the Managing Committee of the Grimsby Hospital the Chairman called attention to the increased quantity of stimulants used for patients during the last six months. The matter was discussed at the meeting, and was then allowed to drop.

The Coventry Hospital Saturday Committee have repudiated the promise of the contribution of £500 towards the extensions at the Coventry and Warwickshire Hospital, stating that they had not authorised the representative who mentioned the matter to make such a promise.

At a meeting of the Swansea Hospital it was resolved that £1,000 be placed at the disposal of the Board of Management for extending the heating of the hospital, which amount, together with £1,500 due on the building account, the annual meeting is to be asked to provide out of capital.

The workpeople at the Pontardawe Steel and Tinplate Works, near Swansea, have subscribed £93 13s. to the Swansea Hospital. To this sum Messrs. Gilbertson and Co., the proprietors of these works, have added £48 8s. 4d., being a donation of £25 and 25 per cent. on the men's contributions, making in all a donation to the hospital of £142 1s. 4d. The workpeople have promised to repeat the contribution in October or November, and Messrs. Gilbertson will in that case repeat their percentage. Such help on the part of the workpeople and the kindly encouragement of their employers is highly commendable.

The past year's collections of the Middlesboro' Hospital Saturday and Sunday Fund amounted to £397 12s. 2d., the expenditure being £10 2s. 2d., leaving £387 2s. to be divided, as against £329 5s. last year. Of the former amount £261 12s. 6d. was derived from the workpeople's collections and £135 5s. 8d. from the church and chapel collections.

During the third quarter of the financial year the average number of patients per day at the Edinburgh Royal Infirmary has been 722, being an increase of 22 as compared with the corresponding period of last year. The total expenditure for the quarter amounted to £10,992, or a net increase of nearly £645. The income amounted to £7,602 3s. 7d.

The total receipts from the Hospital Saturday Committee this year at Tunstall were £363 12s. 5d.; the total expenditure was £28 15s. 2d.; so that the balance distributed was £334 17s. 3d., of which £200 was apportioned to the North Staffordshire Infirmary, £25 to the Llanfairfechan Home, £5 5s. to the Buxton Home, and the balance to the Tunstall Town Nurses' Fund.

Mr. J. Passmore Edwards' offer to build a hospital at East Ham, at a cost of £3,000, is conditional on the Shrewsbury Road site being purchased by the committee. In order to do this £1,500 is needed by September 25th, of which Colonel Burges has promised to give £500. Every possible effort is to be made to collect the necessary sum, so that the purchase can be concluded.

At a recent meeting of the Leicester Guardians it was unanimously resolved, after full discussion, that their scheme to remove the whole of the sick poor, imbeciles, and epileptics from the existing workhouse to an infirmary and special buildings which are to be acquired be accepted; and it was decided to invite architects to submit plans for an infirmary and homes to accommodate 510 sick poor, imbeciles, and epileptics.

### THE STUDENT OF MEDICINE.

### APPOINTMENTS.

W. J. T. Baker, L.R.C.P., L.R.C.S.Edin., L.F.P.S.Glasg., appointed Medical Officer for the South District of the Sheffield Union. T. W. W. Bovey, M.R.C.S.Eng., L.R.C.P.Lond., appointed Medical Officer and Public Vaccinator for the Abbotsbury District of the Weymouth Union, and for the Long Bredy District of the Dorchester Union. F. W. Broadbent, M.B., C.M.Edin., appointed Clinical Assistant to the Chelsea Hospital for Women. E. A. Brown, M.B.Edin., M.R.C.S., L.R.C.P.Lond., appointed Second Assistant Medical Officer to the St. Marylebone Infirmary. A. H. Buchan, M.B., C.M., appointed Clinical Assistant to the Out patient Department of the Victoria Hospital for Consumption. R. McN. Buchanan, M.B., C.M.Glasg., F.F.P.S.Glasg., appointed Bacteriologist to the Corporation of Glasgow. W. Calwell, M.D., M.Ch.R.U.I., appointed Staff Physician to the Belfast Royal Hospital. J. C. Daly, L.R.C.P., L.R.C.S.Edin., appointed Workhouse Medical Officer of the Borrisokane Union. J. Duff, M.D., M.B., C.M., appointed Medical Officer of the Infectious Hospital, Chester. E. T. Ensor, M.D.New York, L.R.C.P.I., L.F.P.S., L.S.A., appointed Medical Officer for the Workhouse for Able-bodied Men and for the Casual Wards Kensington Union. E. G. Ffrench, M.B., Ch.B.Edin., appointed House Surgeon to the Royal Alexandra Hospital, Brighton. D. Fraser, M.B., C.M.Edin., appointed Medical Officer for the First District of the Hawarden Union. F. A. S. Hutchinson, M.R.C.S.Eng., L.R.C.P.Lond., appointed Medical Officer for the Dunmow District of the Dunmow Union. A. P. Lloyd, M.B.Durh., B.S., appointed Medical Officer for the Newbottle District of the Houghton le Spring Union. A. Loxton, F.R.C.S.Edin., appointed Honorary Surgeon to the Birmingham and Midland Hospital for Skin and Urinary Diseases. H. L. McKisack, M.D.R.U.I., appointed Assistant Physician to the Belfast Royal Hospital. C. W. Windsor, M.D., appointed Medical Officer to the Workhouse of the Royston Union. A. M. Watson, M.B., Ch.B., appointed Resident Physician to the Victoria Hospital for Consumption, Edinburgh.

Demy 8vo., over 400 pp., with two Plates, cloth gilt, 12s. 6d. net.

### MEDICAL HISTORY FROM THE EARLIEST TIMES.

By E. T. WITHINGTON, M.A., M.B., Oxon.

"One of the best attempts that has yet been made in the English language to present the reader with a concise epitome of the history of medicine, and as such we very cordially commend it."—*Glasgow Medical Journal.*

London: The Scientific Press, Ltd.,
28 & 29, Southampton Street, Strand, London, W.C.

